# Quantifying normal ankle joint volume: An anatomic study

**DOI:** 10.4103/0019-5413.45326

**Published:** 2009

**Authors:** Reid W Draeger, Bikramjit Singh, Selene G Parekh

**Affiliations:** Kenan-Flagler Business School, University of North Carolina Business School, University of North Carolina School of Medicine, Department of Orthopaedics, Carolina, USA

**Keywords:** Ankle joint, arthroscopy, joint volume, pressure volume, intrarticular injection

## Abstract

**Background:**

Many therapeutic and diagnostic modalities such as intraarticular injections, arthrography and ankle arthroscopy require introduction of fluid into the ankle joint. Little data are currently available in the literature regarding the maximal volume of normal, nonpathologic, human ankle joints. The purpose of this study was to measure the volume of normal human ankle joints.

**Materials and Methods:**

A fluoroscopic guided needle was passed into nine cadaveric adult ankle joints. The needle was connected to an intracompartmental pressure measurement device. A radiopaque dye was introduced into the joint in 2 mL boluses, while pressure measurements were recorded. Fluid was injected into the joint until three consecutive pressure measurements were similar, signifying a maximal joint volume.

**Results:**

The mean maximum ankle joint volume was 20.9 ± 4.9 mL (range, 16–30 mL). The mean ankle joint pressure at maximum volume was 142.2 ± 13.8 mm Hg (range, 122–166 mm Hg). Two of the nine samples showed evidence of fluid tracking into the synovial sheath of the flexor hallucis longus tendon.

**Conclusion:**

Maximal normal ankle joint volume was found to vary between 16–30 mL. This study ascertains the communication between the ankle joint and the flexor hallucis longus tendon sheath. Exceeding maximal ankle joint volume suggested by this study during therapeutic injections, arthrography, or arthroscopy could potentially damage the joint.

## INTRODUCTION

The ankle is a synovial hinge joint that, like any synovial joint, can expand or contract in volume under pathologic conditions such as hemarthrosis or degenerative osteoarthritis, respectively. Many therapeutic or diagnostic modalities require the introduction of fluid into the ankle joint space, including intraarticular injections, arthrography and ankle arthroscopy. The literature currently suffers from a paucity of information regarding the volume of the normal, nonpathologic ankle joint. Knowledge of pressure–volume parameters of normal ankle joints could help improve clinical applications of fluid infusion into the ankle joint. More accurate assessments of maximal normal ankle joint volume could aid orthopedic surgeons in determining optimal volumes of ankle injections to alleviate pain or to visualize the anatomy of the joint without distorting the normal orientation of the contents of the joint capsule.

Several radiographic studies have attempted to characterize the volume of synovial fluid in both normal and pathologic ankle joints through magnetic resonance imaging and ultrasonography.^1^–^5^ However, these studies have generally commented on relative sizes between normal and pathologic ankle joints and have not characterized the actual volume or pressures inside the joints. The purpose of this study was to attempt to measure the volume of the normal, nonpathologic ankle joint through pressure measurements taken on cadaveric human ankle joints with incremental volumes of fluid introduced into the ankle joint.

## MATERIALS AND METHODS

Institutional review board approval was not required for this study because only cadaveric tissue was used to conduct the study.

Nine adult, fresh frozen cadaver ankles were obtained from Science Care Anatomical, Inc. (Phoenix, AZ). After thawing the cadaver ankles to room temperature (approximately 20°C), a 22-gauge needle was introduced into the ankle joint of each of the specimens under fluoroscopic guidance. The needle was connected to a Stryker intracompartmental pressure monitor (Stryker Instruments, Kalamazoo, MI).

**Figure 1:**
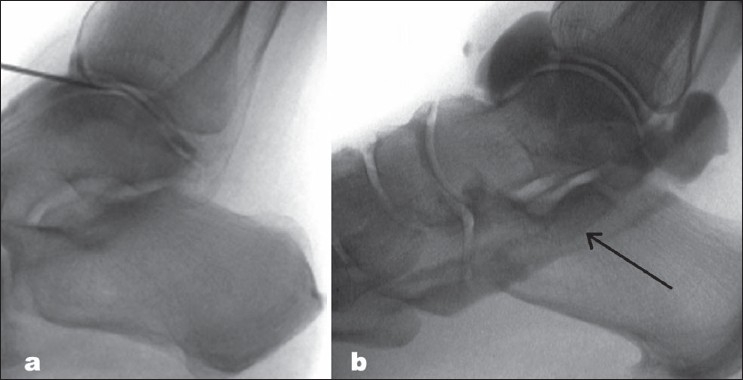
(a) Fluoroscopic image of the needle being introduced into the ankle joint space and confirmed with a small injection of radiopaque dye, (b) Fluoroscopic image illustrating the tracking of some radiopaque dye into the flexor hallucis longus tendon sheath.

After injecting a small amount (approximately 1 mL) of radiopaque contrast [Omnipaque (Iohexol) injection 300 mg/mL, Amersham Health, Inc., Princeton, NJ] into the joint to confirm the placement of the needle into the joint space [Figure 1a], an initial pressure measurement was made. A 10:1 dilution of normal saline to radiopaque contrast was then injected into the joint in 2- mL boluses, and pressure readings were taken on the Stryker device at each of these 2- mL intervals. This solution was injected until significant resistance was met while introducing a bolus, and pressure measurements remained similar from reading to reading, signifying a maximal joint volume, for three consecutive boluses. Fluoroscopic images were taken at various intervals throughout the injection procedure [Figure 1b] to confirm joint space placement of the needle and to ensure the ankle capsule remained intact.

Ankle joint volume and pressure measurements were summarized using routine descriptive statistics (means, standard error).

## RESULTS

Eight of the ankles were left and right foot pairs, and one was a single left foot. Little was known of the medical history of the donors, but they included two males (three ankles, one pair) and three females (six ankles, three pair), ranging in age from 59–73 years (mean age = 66.2 years). None of the donors had any known history of ankle pathology or radiographic evidence of ankle pathology. The heights and weights of these donors varied widely from 145 to 183 cm and 51 to 91 kg, as did the body mass indices (BMI) of the donors (range, 19.2–43.3).

**Figure 2:**
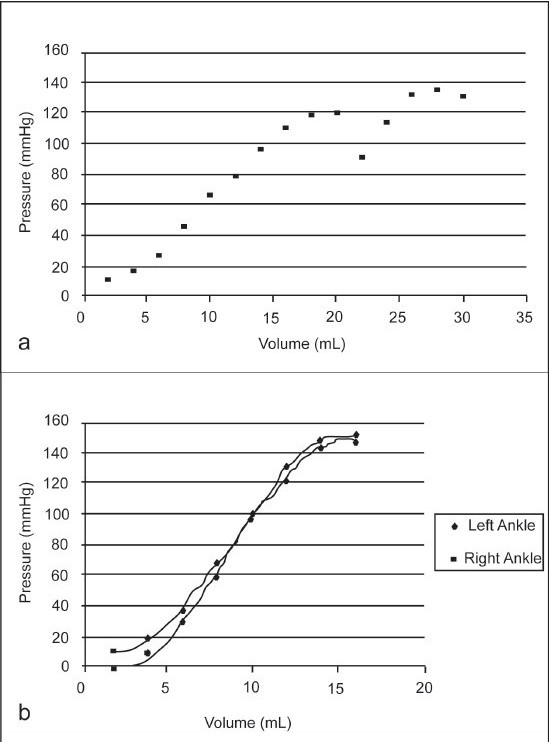
(a) Average ankle joint pressure measurements as a function of volume injected into the joints. The curve approximates an S-shape, suggesting that extremes of volume do not affect ankle joint pressure significantly, (b) A representative plot of the S-shaped pressure–volume curve of a pair of ankles from a single donor. Other donor pairs displayed S-shaped curves, which were similar between feet.

Figure 2 shows the average ankle joint pressure measurements as a function of volume injected into the joints. The mean initial pressure measurement for the nine samples was found to be 9.6 mmHg (range, 3–14 mmHg). The mean maximum ankle joint volume for the nine samples was found to be 20.9 ± 4.9 mL (range, 16–30 mL). The mean ankle joint pressure at maximum volume was 142.2 ± 13.8 mmHg (range, 122–166 mmHg). Two of the nine samples from different donors showed evidence of fluid tracking into the flexor hallucis longus tendon sheath, which has been described, as continuous with the ankle joint in some normal individuals [Figure 2a].^2^,^5^,^6^ The mean volume and pressure measurements at which the S-shaped pressure-volume curve began to increase in a linear fashion were found to be 8.0 ± 2.6 mL (range, 6–12 mL) and 34.0 ± 10.0 mmHg (range, 18–50 mmHg), respectively. The mean volume and pressure measurements at which the S-shaped pressure-volume curve began to plateau were found to be 17.6 ± 5.1 mL (range, 14–26 mL) and 129.1 ± 17.5 mmHg (range, 102–145 mmHg), respectively [Figure 2b].

## DISCUSSION

The ankle joints displayed a nonlinear, S-shaped volume-pressure relationship [Figure 2a]. With low volumes, pressure readings increased slowly, until a more linear part of the curve was encountered. The pressure–volume curve then followed this linear pattern, until the joint began to reach its maximum volume. At this point, there was very little change in pressure as more volume was injected into the joint.

This plateau phenomenon can potentially be explained with a few hypotheses. First, it is possible that after a certain intraarticular pressure is reached, fluid may extravasate from the joint capsule into the soft tissues at approximately the same rate it is introduced into the joint. However, the extravasation of contrast was not observed in our postinjection fluoroscopic images. Another possible explanation for the plateau phenomenon could be that the joint may exceed the elastic recoil capabilities of the joint capsule at such volume and pressure and capsule may become plastically deformed to accommodate each additional increment of volume.

**Figure 3:**
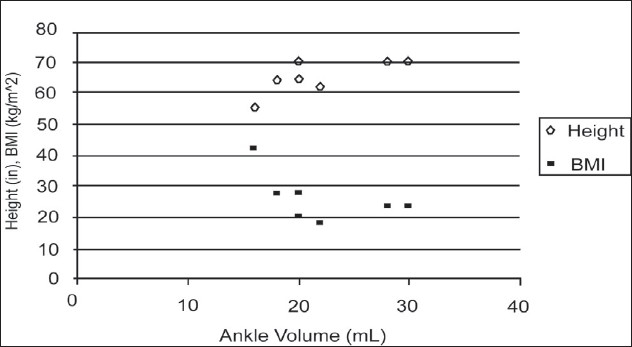
A plot of donor height and BMI versus maximum ankle joint volume suggests a positive relationship between height and maximum ankle joint volume and a negative relationship between BMI and ankle joint volume

Although the small number of observations in this study limits the generalizations that may be made from this data. The maximal ankle joint volume may be directly related to height and indirectly related to BMI [Figure 3]. It could be argued that taller people have a larger ankle bone structure and thus a larger potential space in their ankle joints. Also, people with higher BMIs likely subject their ankle joints to higher forces while weight bearing, which may serve to decrease the potential ankle joint volume over time. It could be hypothesized that when the ankle joint is continually compressed by these excessive forces, the capsule of the joint may contract, limiting its maximal volume.

To our knowledge, there are no previous studies of maximal normal ankle joint volume in the literature. A few studies have tried to characterize synovial fluid volume and distribution in normal as well as pathologic ankle joints that can be visualized with ultrasonography and magnetic resonance imaging.^1^,^2^,^4^,^5^ Through the use of ultrasound, Schmidt *et al* characterized the physiologic normal volumes of the human ankle joint without any fluid being introduced into the joint.^4^ Several studies have characterized normal fluid distribution in the ankle to include not only the joint capsule, but also surrounding bursae and tendon sheaths, especially the flexor hallucis longus and common peroneal tendon sheaths.^2^,^5^ Studies have also found that in some normal ankles there is a communication between the joint space and the surrounding tendon sheaths, a finding which our study confirmed.^2^,^5^,^6^

There are a number of clinical applications where our findings may be pertinent. Intraarticular injections are common both as therapeutic modalities and for arthrographic imaging. Our data suggest that intraarticular injections for either of these purposes should not exceed 16–30 mL. Injections approaching these volumes for therapeutic reasons, such as corticosteroid injections for pain relief, may serve to distend the joint capsule and exacerbate joint pain. Injection volumes of radiopaque contrast agents for arthrograms should remain well under these maximal volumes to avoid patient discomfort from joint capsule distension.

Our findings are also applicable to ankle arthroscopy, in which the surgeon commonly employs large intraarticular volumes of solution to distend the joint for proper visualization. Three commonly referenced orthopedic texts recommend the infusion of 15–20 mL of solution into the ankle joint to aid in joint distension for portal placement, following which, more fluid is infused into the joint.^7^–^10^ Our study suggests that such volumes could lead to extreme pressures in the ankle joint. Importantly, as many of the tendon sheaths of the plantarflexion musculature of the ankle communicate with the ankles of normal individuals,^2^,^5^,^6^ exceeding the maximum ankle joint volumes suggested by our data could lead to increased pressures within these compartments and possibly a compartment syndrome.^11^ Although such a severe complication has not yet been reported for ankle arthroscopy, several authors have described this complication following knee arthroscopy, during which joint pressure and instilled fluid volumes were controlled by infusion pumps or were otherwise poorly monitored.^12^,^13^

Our study has some obvious limitations. First, the study was performed on cadaveric ankle joints. Human cadaveric ankles may behave differently in terms of distensibility and elasticity than the tissues in ankles of living patients. Second, the small number of ankles on which this study was performed limits the generalizability of our results. However, despite its limitations, we believe that this study provides valuable data regarding a subject that is sparsely covered in the literature. Further studies could be conducted in ankle arthroscopy patients to attempt to quantify maximum normal ankle joint volumes in living patients and to attempt to determine the minimal volume of fluid that provides optimum visualization of the ankle joint during arthroscopy. Also, a larger cadaveric or clinical study could be attempted to confirm the relationships of maximum normal ankle joint volume to height and BMI that our results suggest.

In summary, the maximal normal ankle joint volume was found to range from 16 to 30 mL in this study. Infusing such extreme volumes in the joints resulted in high intraarticular pressures, which followed an S-shaped pressure–volume relationship. This volume range could be used to guide the upper limits of fluid infusion into normal ankle joints for procedures such as intraarticular injections, arthrography and ankle arthroscopy.
